# The Emory-Gruentzig Days—Birth of a New Field

**DOI:** 10.1016/j.jscai.2024.102154

**Published:** 2024-07-31

**Authors:** Saraschandra Vallabhajosyula, Spencer B. King, John S. Douglas, H. V. ('Skip') Anderson, J. Jeffrey Marshall

**Affiliations:** a Division of Cardiology, Department of Medicine, Warren Alpert Medical School of Brown University and Lifespan Cardiovascular Institute, Providence, Rhode Island; b Division of Cardiovascular Medicine, Department of Medicine, Emory University School of Medicine, Atlanta, Georgia; c Division of Cardiovascular Medicine, Department of Medicine, McGovern Medical School, The University of Texas Health Science Center, Houston, Texas; d Northside Hospital Cardiovascular Institute, Atlanta, Georgia

**Keywords:** Andreas Gruentzig, Emory University, interventional cardiology, percutaneous transluminal coronary angioplasty

Cardiovascular medicine, and specifically interventional cardiology, has progressed rapidly, with exponential gains in the management of coronary artery disease in the past decades.^1^ Nearly a century ago in Germany, Werner Forssmann performed the first right heart catheterization on himself, demonstrating the feasibility of percutaneously approaching the circulatory system. Subsequently, Cournard and Richards at the Bellevue Hospital in New York described the first diagnostic cardiac catheterization to assess blood loss during the Second World War.^2^ In 1958, Mason Sones at the Cleveland Clinic performed the first coronary arteriogram by accidently injecting contrast media into the right coronary artery, presuming his catheter tip to be in the aortic root.^2^ These pioneering achievements paved the way for catheter-based interventions for coronary artery disease and formed the bedrock for planning minimally invasive coronary interventions.^3^

Andreas Gruentzig is the uncontested father of interventional cardiology.^4^ He completed medical school in 1964 and went on to pursue clinical, research, and laboratory work in angiology, peripheral vascular disease, statistics, and epidemiology in Germany, Switzerland, and the United Kingdom.^2^^,^^4^ In 1971, he attended a lecture where Eberhardt Zeitler, another angiologist and radiologist, described Charles Dotter’s work in peripheral arteries. Dotter used concentric telescoping catheters that could be passed in sequence, smaller to larger, through an obstructing lesion, pushing aside or compressing the atherosclerotic plaque material and reducing the degree of stenosis.^4^ Gruentzig sought to adopt and refine Dotter’s balloon angioplasty technique for vascular applications. He worked closely with Heinrich Hopff at the Swiss Federal Polytechnic Institute to develop polyvinyl chloride-based balloon dilatation catheters^2^^,^^4^ and started miniaturizing and improvising them to make them suitable for coronary use. After a series of experiments in dogs, on September 16, 1977, Gruentzig performed the first human percutaneous transluminal coronary angioplasty (PTCA) at the Zurich University Hospital.^4^ This was the first of many successful angioplasty cases and the birth of a new field.

In 1980, Gruentzig moved to Emory University in Atlanta to work alongside Spencer B. King III and John S. Douglas. In those pioneering years, the Emory University Cardiac Catheterization Laboratory (CCL) was a hub of activity and innovation. Stiff Teflon guides and balloon-tipped double-lumen PTCA catheters were refined, and steerable guide wires and over-the-wire PTCA catheters led to dramatic improvements in the success rates of PTCA at Emory University.^2^ In about 1985, Cesare Gianturco, a retired radiologist, introduced his prototype vascular stent sized for coronary arteries and brought it to Gruentzig.^4^ Gruentzig assigned his senior fellow, Gary S. Roubin, to perform the preclinical testing on it.

In this issue of *JSCAI*, we launch our new video series Pioneers in Interventional Cardiology*,* featuring Dr King and Dr Douglas recounting their early experience at the Emory CCL. Dr Douglas describes his experience putting in the first coronary stent in 1987 as a bailout for a dissected left anterior descending. “Stents were not quite used for the same purposes as today,” Dr King points out. “They were used to deal with acute vessel closure in the CCL. Before stents, these patients had to go urgently to the operating room for emergency coronary artery bypass surgery.” Dr King reminisces about the days when they did not have fluoroscopic videos but had to use fluoroscopic road maps. “…A single image intensifier was used to draw diagrams, to help ‘roughly’ guide our work.” However, not all inventions and discoveries paid off. “Andreas insisted that we get him an isocentric biplane. He thought this was going to be crucial. We bought that thing, and it spent more in the shop than anywhere else!” King remembers.

In parallel with the early advances of PTCA, Gruentzig, King, and Douglas had the incredible foresight to establish evidence with a strong research program to quantify this cumulative knowledge. They, in collaboration with others, contributed to the development of the National Heart, Lung, and Blood Institute PTCA registry, and all cardiologists performing PTCA at that time were required to enroll patients. The popularity of the PTCA technique increased the clamor for additional learning and teaching. This resulted in the institution of the popular Emory demonstration courses that spurred the dissemination of the latest techniques and equipment to the interventional cardiology community. Talking about the course, King recalls, “We had these 2 10-foot back-project TV images that were called ‘hi-def’ in those days…nothing like high definition now. To get that, we ran a fiber optic cable from the cath laboratory to the Woodruff building to project the fluoroscopic images and the people working in the lab.” These case demonstrations formed the bedrock of live courses that continue to educate interventional cardiologists in the contemporary era and have inspired so many. King and Douglas describe experiences during these live cases that were quite entertaining, albeit not for the operator. King describes their early experiences with proctoring and apprenticeship among fellows and other practicing physicians that helped grow the field of interventional cardiology. These relationships grew more robust and improved the referrals to the Emory University Hospital. Douglas states, “Colleagues around the state had cases that could be done but didn’t want to do it in their hospital, so they would bring it to us. It gave us the opportunity to see these difficult cases and to use new technologies such as rotational atherectomy, directional atherectomy, and stents.” Douglas shared his vast experiences with PTCA of coronary artery bypass grafts, the neointimal hyperplasia, and selecting the right vein graft to consider for PTCA.

King and Douglas recount their early experiences at the Emory CCL fondly. Douglas remembers, “…there was a sense of camaraderie! The equipment was pretty crude back in those days. You would expect to have 5% of the cases not turn out very well. It was pretty tense in those days.” King states, “A major part of our practice was how to get out of trouble because you got into trouble so often!” When asked how the relationship was with the cardiac surgeons, they said, “We had a history with the surgeons. We had supported them, but at the same time we started doing angioplasty and were having all these failures. They were not completely happy with that. Andreas had gotten to know not only us [the interventional cardiologists] but also the surgeons. He was under so much pressure in Zurich where the people in charge were not happy with what he was doing. The reason he came to Emory, in part, had to do with he felt more comfortable dealing with us and with the surgeons than he had where he had been.” Douglas recalls, “The anesthesia group was extremely talented. They recruited lots of folks…who were just superb.” The critical role for multispecialty collaboration and the “heart team” model advocated for in the current literature was foundational to the success of the early PTCA program at Emory.^5^ The volumes started to grow rapidly in those days, and quickly Gruentzig had to go to the Crawford-Long Hospital (currently Emory University Hospital Midtown) to do peripheral cases and surplus coronary cases. This longstanding relationship between the 2 hospitals continues as a large, single, integrated cardiology division.

In closing, Douglas and King share their insights on work-life balance…or lack thereof. Douglas notes, “We would show up early in the morning and get home late in the evening, and our wives would be beating on us for missing supper! We were blessed with these terrific young people, fellows, nurses and staff, it was an awesome experience. My wife kept telling me you are crazy…you need to reset your priorities!” They discuss Gruentzig’s untimely death at a young age, the value of life and relationships, and the importance of being a dedicated doctor. These principles are truly the legacy of Gruentzig and Emory University to the young interventional cardiologists who want to follow in their footsteps.
